# A single arm phase II study of bone-targeted Sn-117 m-DTPA in symptomatic castration-resistant prostate cancer with skeletal metastases

**DOI:** 10.1186/s12885-022-09496-2

**Published:** 2022-04-15

**Authors:** Zin W. Myint, Riham El Khouli, Bryan Lemieux, Donglin Yan, William H. St. Clair, Xiaoqi Liu, Charles A. Kunos

**Affiliations:** 1 Department of Medicine, Division of Medical Oncology, University of Kentucky, Lexington, KY 40536 USA; 2 Markey Cancer Center, University of Kentucky, Lexington, KY 40536 USA; 3 Department of Nuclear Medicine, University of Kentucky, Lexington, KY 40536 USA; 4 Department of Radiation Safety, University of Kentucky Health Care, Lexington, KY 40536 USA; 5 Department of Radiation Medicine, University of Kentucky, Lexington, KY 40536 USA; 6 Department of Toxicology and Cancer Biology, University of Kentucky, Lexington, KY 40536 USA

**Keywords:** Metastatic castration resistant prostate cancer with bone met, Bone pain, Bone seeking radionuclides, Pain response, Analgesic consumption, Patient reported outcome

## Abstract

**Background:**

Several bone-seeking radionuclides have been developed for palliation of metastatic bone pain since 1956, however, so far radium-223 dichloride is the first and only Food and Drug Administration (FDA) approved targeted alpha therapy for metastatic castration-resistant prostate cancer (mCRPC) based on ALSYMPCA phase 3 study. While radium-223 does improve pain and overall survival outcomes, the improvement can come at the expense of side effects such as bone marrow toxicity. The development of new and better treatment with long-standing pain relief is clearly an unmet medical need.

**Methods:**

The study is a non-randomized phase II study. The study population consists of 25 patients with CRPC who had progressed on any lines of prior therapies and whose serum testosterone level is less than 50 ng/dl and have metastatic lesions to at least two bone sites, with at least one site that has clinically meaningful pain at baseline (≥ 4 on an 11-point intensity scale). Eligible patients will be given two cycles of Sn-117 m-DTPA every 8 weeks or 56 days. Treatment will be administered by slow IV injection over 5–10 min. Retreatment after two cycles is allowed if patients meet the following retreatment criteria. The primary objective is to evaluate the efficacy of Sn-117 m-DTPA on sustained pain response in patients with CRPC metastatic to at least two bone sites and at least one with clinically meaningful pain at baseline (≥ 4 on an 11-point pain intensity scale). Sustained pain response is defined as: 1) achieving pain index ≤ 3 within a 12-week period and 2) maintaining pain index ≤ 3 over a 16-week period. The secondary objectives are: safety and tolerability, measurement of Sn-117 m-DTPA activity by gamma-camera dosimetry scans, therapeutic efficacy, time to the first symptomatic skeletal event, duration of pain response, changes in PSA and ALP levels, patient-reported outcomes and progression free survival and overall survival.

**Discussion:**

Sn-117 m-DTPA is a unique bone-targeting theranostic radiopharmaceutical agent that selectively binds most heavily to bone metastases sites. This study will be the first prospective phase II trial to assess the pain efficacy and anti-tumor activity of Sn-117 m-DTPA in mCRPC with at least one clinically meaningful pain at baseline.

Trial registration.

ClincialTrials.gov Identifier: NCT04616547.

## Background and rationale

More than 80% of advanced prostate cancer patients develop bone metastases [1], and often experience unpleasant bone pain in their cancer journey. Current understanding of cancer-induced bone pain involves multi-complex mechanisms including neuropathic, inflammatory, ischemic, and cancer specific pathways [2]. The intensity of cancer bone pain can be highly variable and pain management usually involves a combination of palliative radiation therapy in addition to large quantities of narcotics. A single-fraction, high-dose stereotactic body radiation therapy (SBRT) was effective in relieving cancer bone pain from any solid tumors in a prospective clinical trial [3]. Improvement in overall pain response as measured by combination of patient-reported pain score and narcotic use at 2 weeks, 1 month and 3 months were reported as 62%, 44% and 38% respectively [3]. However, SBRT treats a limited number of bone metastases at any one time and may not be suitable to treat in patients with extensive bone metastases inducing pain.

Several bone-seeking radionuclides have been developed for palliation of metastatic bone pain since 1956 [4–6]. Among these, phosphorous-32 [6], strontium-89 [7, 8], samarium-153 [9, 10], and rhenium-186 [11, 12] are all beta-emitters and radium-223 dichloride [13] is an alpha particle-emitter. The unique differences between beta-emitter, alpha-emitter and the study drug are described in Table 1. So far radium-223 dichloride is the first and only Food and Drug Administration (FDA) approved targeted alpha therapy for metastatic castration-resistant prostate cancer (mCRPC) based on ALSYMPCA phase 3 study [13]. The pain response was evaluated after a single injection of different doses of radium-223 (5, 25, 50 or 100 kBq/kg) in a phase 2 double-blind study [14]. Pain response was assessed by self-assessment of pain score on visual analogue scale and patients recorded analgesic consumption. It showed statistically significant improvement in reported pain response as 40%, 63%, 56%, and 71% pain responders for the 5, 25, 50, 100 kBq/kg groups respectively at week 8. The mean pain relief duration was 44 days in the 50 and 100 k Bq/kg groups [14]. While radium-223 does improve pain and overall survival outcomes, the improvement can come at the expense of side effects such as bone marrow toxicity. The development of new and better treatment with long-standing pain relief is clearly an unmet medical need.

**Table 1:** Unique differences between bone-seeking radionuclides

	Beta-emitter	Alpha-emitter	Study drug (117 m-Sn-DTPA)
Characteristics	High-energy, high-speed electron (variable energy and penetrance)	Highly localized (short range), high-linear energy transfer	Low energy conversion electron emitter (monoenergetic and finite penetrance)
Range in tissue (µm)	50–5000	40–90	300
Bio-distribution		-Bone uptake 52% (41–57%) -Rapid disappearance from soft tissue -Excrete through small bowel	-Bone uptake 82% and resides in cortical bone rather than adjacent bone marrow cavity -Rapid disappearance from soft tissue -Excrete through kidney
Side effects	Greater bone marrow toxicity	Moderate bone marrow toxicity	Least bone marrow toxicity
Decay	Beta decay	Gamma	Gamma (159 keV)
Half-life	1.9 days (^153^Sam), 14.3 days (^32^ P) and 50.5 days (^89^Sr)	11.4 days	14 days

Sn-117 m-diethylenetriaminepentaacetic acid (DTPA; stannic pentetate injection) is a radiopharmaceutical agent being developed for treatment of malignant bone metastases for any solid tumors including prostate, breast, and lung, starting in the 1980s by Srivastava and colleagues at Brookhaven National Laboratory [15]. Sn-117 m-DTPA is a radioactive isotope of tin that is a low-energy conversion electron emitter (127 keV, 129 keV, 152 keV, and 140 keV) and also yields a gamma emission of 159 keV [16]. The biodistribution of Sn-117 m-DTPA was studied in animal models using whole body autoradiography which showed that Sn-117 m-DTPA had high affinity to normal bone with low soft tissue concentration [17]. Sn-117 m-DTPA also displayed uptake autoradiographically in human osteosarcoma xenografts in nude (athymic) mice [17]. Atkins et al. studied the whole-body distribution of Sn-117 m-DTPA and showed that more than 50% of the administered activity was absorbed in the bones of patients with metastatic cancer [18]. The red marrow absorbed dose was low compared with the bone surface dose. All other tissues received less than 1/10 of the dose received by red marrow.

A pilot study of 15 patients with painful skeletal metastases from myriad solid tumors evaluated treatment with Sn-117 m-DTPA at 71–143 mcCi/kg [19]. Results demonstrated pain relief without inducing bone marrow toxicity. Krishnamurthy et al*.* studied the biokinetics and imaging characteristics of Sn-117 m-DTPA radioactivity in 17 patients with metastatic bone pain [20]. Three dose levels were administered IV: 180 mcCi/kg; 229 mcCi/kg; and 285 mcCi/kg of patient body weight. Pain palliation was observed with all three doses, with responses in 60–83% of cases. The duration of pain palliation ranged from 3 to 14 months from a single administration of Sn-117 m-DTPA. The majority (59%) of the administered Sn-117 m-DTPA was taken up in bony metastatic lesions and peaked in three to seven days, whereas peak uptake in normal bones occurred by 24 h. The remaining radioactivity was excreted by the urinary tract (37%) [20].

In a phase 1/2 study, 47 patients with painful bone metastases from a variety of malignancies (including 30 patients with prostate cancer) were treated with five different levels of Sn-117 m-DTPA radioactivity [21]. The dose levels, based on kg of body weight, were as follows: 2.64 MBq (71 mcCi); 5.29 MBq (143 mcCi); 6.61 MBq (179 mcCi); 8.46 MBq (229 mcCi); and 10.58 MBq (286 mcCi). Approximately 45% of patients obtained reduction of pain by at least 50%, and 30% experienced complete relief of pain for more than two weeks [21]. There was no correlation between response rate and the dose levels. However, the time to onset of pain relief was shorter (5 ± 3 days) with doses ≥ 12.5 mCi/70 kg than with doses ≤ 10 mCi/70 kg (19 ± 15 days) [21].

Collectively, there is mounting evidence that Sn-117 m-DTPA has promising palliative efficacy with minimal toxicity. Thus, a focused evaluation of this agent in patients with bony metastases is urgently required. The primary objective is to evaluate the efficacy of Sn-117 m-DTPA on sustained pain response in patients with CRPC metastatic to at least two bone sites and at least one with clinically meaningful pain at baseline (≥ 4 on an 11-point pain intensity scale). Sustained pain response is defined as: 1) achieving pain index ≤ 3 within a 12-week period and 2) maintaining pain index ≤ 3 over a 16-week period.

Secondary objectives include: 1) assessment of the safety and tolerability by Sn-117 m-DTPA per Common Terminology Criteria for Adverse Events (CTCAE) v.50; 2) measurement of Sn-117 m-DTPA activity by gamma-camera dosimetry scans (serial full body planar images) at 1 h, 4 h(, 24 h(, 48 h(, 72 h(, 1 week( and 4 weeks ( after the first Sn-117 m-DTPA administration; 3) evaluation of the therapeutic efficacy of Sn-117 m-DTPA at 24 weeks as measured by Prostate Cancer Working Group 3 (PCWG3) criteria; 4) evaluation of time to the first symptomatic skeletal event defined as i) the first use of external-beam radiation therapy to relieve skeletal symptoms; ii) new symptomatic pathologic vertebral or nonvertebral bone fractures; iii) spinal cord compression; or iv) tumor-related orthopedic surgical intervention; 5) to evaluate the overall pain response rate at 24 weeks; 6) to evaluate the duration of pain response as defined from the time of improvement in pain response (pain index ≤ 3) until the pain recurs; 7) to measure changes and time to progression in serum prostate-specific antigen (PSA) and serum alkaline phosphatase (ALP) levels; 8) to assess patient-reported outcomes (PROs) and adverse events (AEs) (PRO-CTCAE) captured by digital instruments; 9) to evaluate progression-free survival (PFS) and overall survival (OS).

## Methods

**Fig. 1:**
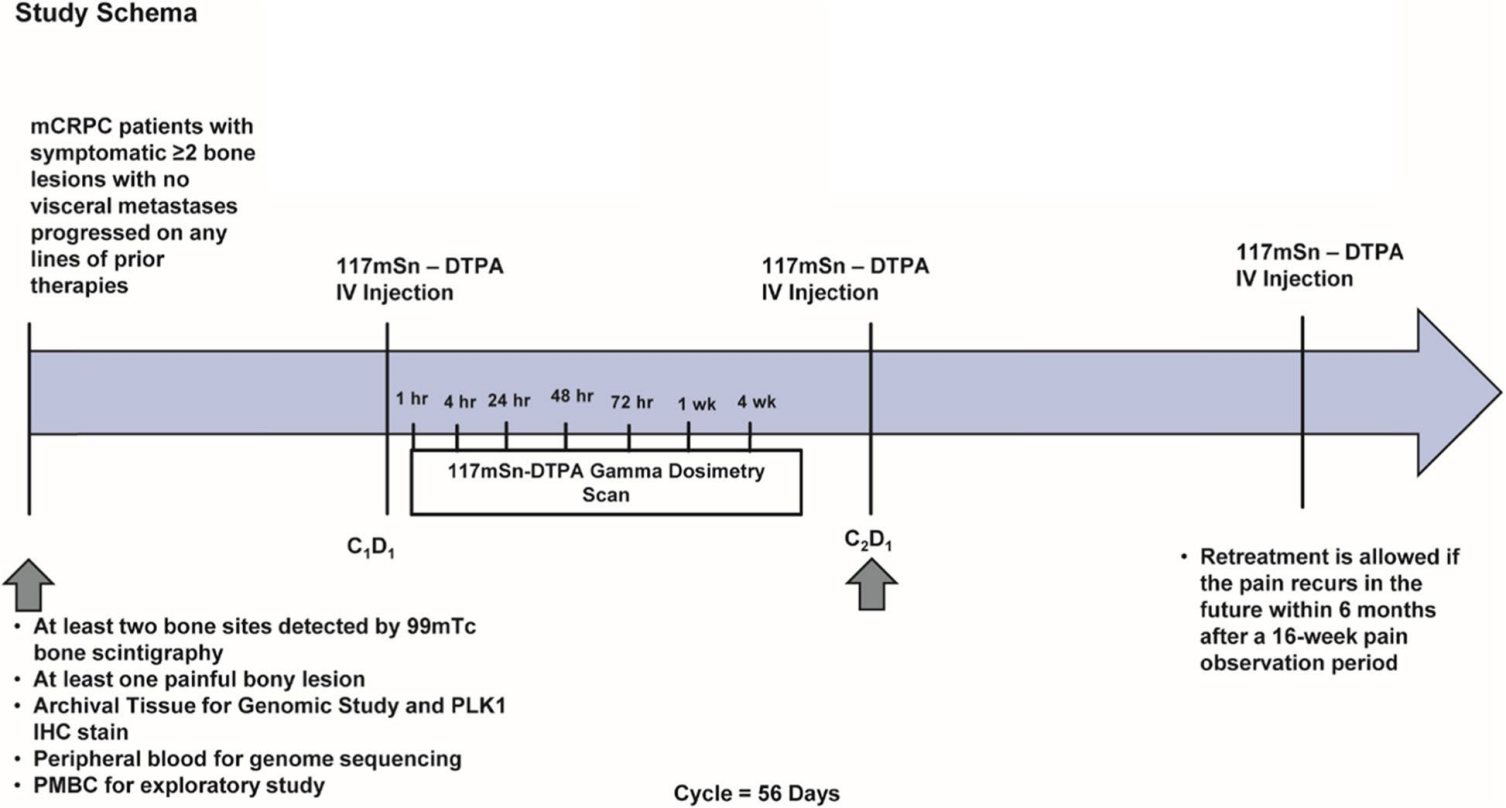
Study Schema

The present study is a non-randomized phase II study. This study aims to compare the efficacy and safety of Sn-117 m-DTPA as a pain palliative agent in patients with symptomatic CRPC metastatic to at least two bone sites detected by 99Tc bone scintigraphy with at least one site with clinically meaningful pain at baseline (≥ 4 on an 11-point pain intensity scale). The efficacy and safety of Sn-117 m-DTPA will be evaluated, and the effect of therapy on pain palliation will be investigated. Ethics approval is obtained at central IRB. The trial is registered at clinicaltrial.gov (NCT04616547). The study schema is shown in Fig. 1.

The study population consists of 25 patients with CRPC who had progressed on any lines of prior therapies and whose serum testosterone level is less than 50 ng/dl and have metastatic lesions to at least two bone sites, with at least one site that chronicles clinically meaningful pain at baseline (≥ 4 on an 11-point intensity scale).

### Key Inclusion criteria

- Patients must have histologically or cytologically confirmed adenocarcinoma of the prostate that is castration-resistant, defined as: i) a castrate serum testosterone level ≤ 50 ng/dL or 1.7 nmol/L; ii) bilateral orchiectomy or maintenance on androgen ablation therapy with luteinizing hormone-releasing hormone (LHRH) or antiandrogen such as bicalutamide; androgen deprivation therapy needs to be maintained throughout the study unless a patient has had orchiectomy by surgery; iii) serum PSA progression defined as two consecutive increases in PSA over a previous reference value, each measurement at least 1 week apart.
- Progression after androgen receptor blockers (enzalutamide, apalutamide, or darolutamide) or androgen synthesis blockers (abiraterone acetate) or chemotherapy (docetaxel or cabazitaxel). There are no maximum number of prior therapies.
- Progressive castration-resistant prostate cancer with two or more skeletal metastases identified by Tc-99 m bone scintigraphy.
- Patients must have self-reported moderate to severe pain at trial entry (baseline weekly average “worst pain in the past 24-h” scores of ≥ 4 on an 11-point numeric rating scale [NRS], the Brief Pain Inventory – Short Form [BPI-SF] item #3 for worst pain).
- Patients must either currently employ regular (not occasional) analgesic medication use for cancer-related bone pain or have undergone treatment with external beam radiation therapy for bone pain within 12 weeks before starting study treatment.

### Key exclusion criteria

- Patients must not have visceral metastases (such as liver and lung) as assessed by abdominal/pelvic CT or chest X-ray within 12 weeks before starting study treatment.
- Patients must not have malignant lymphadenopathy exceeding 3 cm in short-axis diameter.
- Patients must not have imminent or established spinal cord compression based on clinical findings and/or MRI.
- Patients who have had chemotherapy, immunotherapy, or external radiotherapy within 4 weeks prior to entering the study are excluded.
- Patients must not have received systemic radiotherapy with radium-223, strontium-89, samarium-153, rhenium-186, or rhenium-188 for the treatment of bony metastases within 24 weeks before starting study treatment.
- Patients must not have unmanageable urinary incontinence.
- Patients must not have had known non-pathological bone fractures within 2 months before starting study treatment.

Eligible patients will be given two cycles of Sn-117 m-DTPA every 8 weeks or 56 days (Table 2). Treatment will be administered by slow IV injection over 5–10 min. Sn-117 m-DTPA solution should not be diluted or mixed with any other solutions. The IV access line must be flushed with isotonic saline before and after injection of Sn-117 m-DTPA on an outpatient basis as below. The study drug will be provided by NCI CTEP in collaboration with Serene, LLC. The study drug should be administered only by authorized persons in designated clinical settings. Radiation protection precautions must be taken in accordance with national and local regulations. Sn-117 m-DTPA must be added to a site’s radioactive material license and document training for administration and handling of the agent.

**Table 2:** Study Regimen Description

Agent	Dose	Route	Schedule	Cycle Length
Sn-117 m-DTPA	20 mCi/70 kg (0.28 mCi/kg)	IV injection over 5–10 min	Day 1	8 weeks*

*Retreatment after two cycles is allowed if patients meet the following retreatment criteria: Pain (≥ 4 on 11-point intensity scale) recurs within 6 months after a 16-week pain observation period and no disease progression on bone scans (≤ 2 new bone lesions), or evidence of clinical progression (such as development of cancer-related symptoms). The maximum treatment doses per patient is four injections in this study (2 rounds of 2 injection cycles)

Patients will be instructed to maintain a high fluid intake (drink at least two to three quarts of fluid every 24 h) for 2 weeks after each Sn-117 m-DTPA treatment to minimize a concentrated radioactive excretion. In general, drinking alcoholic beverages should be kept to a minimum or avoided completely. Patients will be on hygiene instruction/precautions on post-therapy treatment.

### Data collection

Patients will be followed every 2 weeks during treatment period (16 weeks), then every 4 weeks until week 28, and then every 3 months or as clinically indicated for 12 months after the first Sn-117 m-DTPA dose or until death, whichever occurs first. After a 1-year follow-up period, dates of death will be collected for all patients until the final patient completes his 1-year follow-up. Patients removed from study for unacceptable adverse event will be followed until resolution or stabilization of the adverse event. The following investigations should be performed at every follow-up visit: 1) pain medication; 2) patient-reported pain intensity scale and analgesic use; 3) patient-reported CTCAE; 4) urine collection for urinalysis; 5) PSA; 6) CBD with diff and serum chemistry. (Table 3).

**Table 3:** Data Collection Schedule

	Pre-study	Cycle 1 (8 weeks or 56 days)					Cycle 2 (8 weeks or 56 days)					Post-treatment						FU^d^	OSV
		W1	W2	W4	W6	W8	W9	W10	W12	W14	W16	W18	W20	W22	W24	W26	W28		
Sn-117 m-DTPA		**A**					**A**											**A**	
^a^Pain medication	X	X	X	X	X	X		X	X	X	X		X		X		X	X	X
CBC/diff, platelets	X	X	X	X	X	X		X	X	X	X		X		X		X	X	X
Serum chemistry	X	X	X	X	X	X		X	X	X	X		X		X		X	X	X
PSA	X			X		X			X		X		X		X		X	X	X
Patient-reported pain intensity scale and analgesic use^b^	X		X	X	X	X		X	X	X	X	X	X	X	X	X	X	X	X
Patient-reported CTCAE^c^			X	X	X	X		X	X	X	X	X	X	X	X	X	X	X	X
Urine collection for urinalysis	X	X	X	X		X			X		X		X		X		X	X	

*A* indicates radiotherapy; *FU* Follow-up (every 3 months), *OSV* Off-Study Visit, *CTCAE* Common Terminology Criteria for Adverse Events, *PSA* Prostate-specific antigen

^a^Study coordinator/research nurse will inquire about pain medications including names, dosage and frequency; this person also is required to fill out pain medication list form during every clinic visit

^b^Pre-treatment daily baseline pain and analgesic use for 7 days (at least 4 out of 7 days) is required before treatment initiation. Patients will be asked to report their pain at its worst in the last 24 h, and analgesic use (stable/reduced/increased) by digital version every 2 weeks through Week 28, followed by routine clinic follow up or when they experience new pain during off-visit. A full short version of brief pain inventory (BPI) will be asked at baseline, after Cycle 1, after Cycle 2, and at Week 24

^c^PRO-CTCAE survey items will be assessed by digital instruments. Beginning at Week 28, surveys are requested every 3 months for 6 months after the last study treatment administration

^d^Follow-up visit evaluation. Every 3 months or as clinically indicated up to one year from the first dose injection or disease progression

### Method of pain assessment

**Fig. 2:**
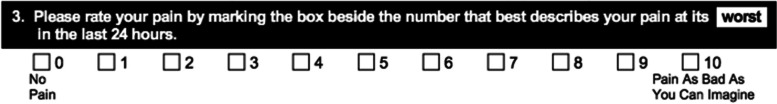
Pain Intensity scale (Item#3 on the BPI-SF, worst pain)

The study coordinator and investigators will monitor adherence by reviewing questionnaires via electronic or paper. Patient-derived information obtained will include: 1); a pain intensity scale (Fig. 2); analgesic use consumption (Table 4).

**Table 4:** Patient-reported analgesic log

Decrease	Stable	Increase

Both narcotic and non-narcotic analgesic use and pain intensity scales will be captured using pain score and analgesic log electronically or a paper format.

The Sn-117 m-DTPA palliative pain effect will be measured by using a pain index as described in the Table 5.

**Table 5:** Classification of pain index based on diary pain intensity rating and analgesic intake

Pain response	Pain index	Diary pain rating change from baseline	Analgesic intake compared with baseline
Complete	1	Decrease ≥ 90%	Stable or reduced
Marked	2	Decrease ≥ 50% to < 90%	Stable or reduced
Moderate	3	Decrease > 33% to < 50%	Stable or reduced
Minimal	4	Decrease ≥ 20% or increase < 33%	Stable
None	5	Decrease < 20% or increase < 20%	Stable
Pain progression	6	Increase ≥ 20%	Stable or increased
		Decrease < 20% or increase < 20%	Increased
		Decrease ≥ 20%	Increased

This pain index is derived from a combination of the following 11-piont pain intensity scale and analgesic consumption categorized according to the World Health Organization analgesic ladder.

### Timing of assessment

Pain at baseline and analgesic use (pre-treatment, week 0) will be measured via serial (daily) assessments in a 7-day run-in period. Patients will be considered evaluable if they complete assessments on ≥ 4 days within the 7-day run-in period. During treatment, pain and analgesic use will be measured every 2 weeks through week 28, followed by routine clinic follow-up until 1 year or when patients experience new pain. Complete pain assessments via the full BPI-SF will be collected at baseline, after cycle1, after cycle2, and at week 28. Pain assessments will be halted at the patient’s request or if the patient discontinues study treatment for reasons other than disease progression.

### Statistical methods

This is a single-arm, open-label phase 2 study to evaluate the efficacy of Sn-117 m-DTPA on sustained pain response in patients with CRPC metastatic to at least two bone sites. Sustained pain-response is defined as: 1) achieving pain index ≤ 3 within a 12-week period and 2) maintaining that pain index ≤ 3 over a 16-week time period (beginning when the pain index ≤ 3 is initially achieved). The maximum duration of follow-up required to declare a patient a responder is 28 weeks, representing a patient who initially achieves pain index ≤ 3 toward the end of the 12-week period and is followed for an additional 16 weeks to confirm maintenance of pain index ≤ 3.

Eligible patients will be assigned to receive Sn-117 m-DTPA at 20 mCi/70 kg (0.28 mCi/kg) every 8 weeks for two cycles. The study utilizes a mini-max Simon two-stage design to assess anti-tumor activity and pain benefit of Sn-117 m-DTPA.

The sustained pain response rate under null hypothesis is p_0_ = 0.1 and the alternative hypothesis is p_1_ = 0.3. The literature on sustained pain-relieving benefit with radiopharmaceutical drugs in prostate cancer with bone metastases is limited. Nilsson et al. studied the dose–response relationship and pain-relieving effect of radium-223 [8]. Pain response at week 8 was seen in 40% of patients. The null hypothesis for sustained pain response in the absence of effective therapy and a strong literature support would assume 10%. We propose that the sustained pain response with Sn-117mDTPA would achieve 30%. Using the mini-max Simon’s two-stage design, the total sample size is 25 patients, with 10 patients for the first stage and 15 patients for the second stage. If 1 or 0 patients achieve sustained pain response in the first stage, the study will be stopped early for futility; otherwise the trial continues to treat with the second stage. If 5 or fewer patients achieve sustained pain-response, then no further investigation of the study treatment is warranted. This design has an estimated 5% type I error rate and 80% power. The planned sample size and interim analysis has a 3.28% actual type I error rate and 80.17% actual power. The probability of stopping at the end of the first stage is about 55% under the null hypothesis. An interim analysis for efficacy will be conducted after the first 10 patients have become evaluable for the primary endpoints. Study enrollment will be interrupted for the interim efficacy analysis.

The efficacy of the primary endpoint will also be summarized by the point estimation of the overall response rate with the corresponding 95% confidence intervals. Patients who received any amount of study drug will be included in the denominator for the calculation of ORR.

Secondary endpoints will be summarized depending on data type. Categorical endpoints will be summarized by frequency and percent. Continuous variables will be summarized by mean and standard deviation at each assessment time point. Time-to-event endpoints will be summarized by Kaplan–Meier Methods. Safety and toxicity will be assessed through the frequency and percent of AEs, serious adverse events (SAEs), and adverse events of special interests. Toxicity will be assessed by clinical staff using (the CTCAE and also by patients [PRO-CTCAE]) captured by digital instruments during treatment and 8 weeks after the last injection to monitor for acute toxicity and every 2–4 weeks for 6 months post-therapy for monitoring of long-term toxicity. CTCAE grades for the corresponding time period will be presented in conjunction with PRO-CTCAE scores. PSA and ALP response rates will be calculated for each of the above defined categories with the corresponding confidence intervals. *Post-hoc* analysis on PSA and ALP levels may be conducted to explore possible trends in PSA and ALP levels. Time to the first symptomatic skeletal event will be analyzed using the Kaplan–Meier method. Estimation and confidence intervals for the median time to first symptomatic skeletal event will be provided. Patients who did not have a symptomatic skeletal event will be censored at the last study visit. Progression free survival will be analyzed in a similar manner as the time to first skeletal event. Patients who did not have observed clinical progression will be censored at the last assessment.

### Adverse events of special interest

Grade 3 or higher renal and genitourinary AEs will be reported as expedited AEs, including but not limited to: 1) renal failure (ranging from significantly reduced measured or estimated creatinine clearance to clinically overt renal failure other than that of obvious non-investigational agent-induced origin; 2) suspected radiation nephropathy of any type, such as radiation-induced thrombotic microangiopathy, manifesting as proteinuria, hypertension, edema, anemia and decreased serum haptoglobin; 3) general symptoms and signs of acute radiation toxicity, such as increased frequency/urgency of urination, nocturia, dysuria, bladder spasm, bladder obstruction, genitourinary ulceration of necrosis; 4) grade 3 or higher bone marrow toxicities will also be reported as expedited AEs.

### Data collection, managing and monitoring

Medidata Rave is a clinical data management system being used for data collection for this study. Access to the trial in Rave is controlled through the CTEP-IAM system and role assignments. All data will be monitored by the Clinical Trials Monitoring Service (CTMS). Data will be submitted to CTMS at least once every two weeks via Medidata Rave. Onsite audits will be conducted by the CTMS. The PI, investigators and statistician will have access to all the data for analysis.

## Discussion

Sn-117 m-DTPA is a unique bone-targeting theranostic radiopharmaceutical agent, that selectively binds most heavily to bone metastases sites. Unlike Radium-223, an alpha-emitter, Sn-117 m-DTPA releases mono-energetic conversion electrons of ~ 140 keV discrete energy for therapy with an average range of ~ 300 µm in tissue penetrance. Therefore, Sn-117 m-DTPA effectively treats only the targeted tissue and reduces the external radiation hazard. Like Radium-223, it yields gamma emission (159 keV) similar to Tc-99 m (140 keV) allowing for existing standard gamma camera imaging and techniques. Biodistribution of Sn-117 m-DTPA in animal models indicates high uptake in cortical bone, and approximately tenfold more radioactivity than in bone marrow. Thus, it appears to have less bone marrow toxicity compared to Radium-223. Sn-117 m-DTPA has a slightly longer half-life (14.5 days) and thus it can be given every 8 weeks.

The previous phase 1/2 dose-ranging study evaluated the effects of Sn-117 m-DTPA administration on pain relief using daily 5-point numeric pain rating scales in all solid tumors after one injection [20, 21]. About one quarter of all patients entered had complete pain relief and an additional one third had > 50% relief of pain. More striking was a decrease in time to onset of pain relief, with a mean time to onset of pain relief of 22 days at the lowest dose, plateauing at 5 days for doses of 12.5 mCi/70 kg and above. Mean duration of response was 3.3 months, with a maximum of 13.4 months. Nether radiologic time to progression nor overall survival was assessed in that study [20, 21]. Results of previous clinical studies concluded that Sn-117 m-DTPA was well tolerated. Four patients had grade 3 neutropenia, however, investigators attributed neutropenia was from chemotherapy after Sn-117 m-DTPA. No grade 3 anemia or thrombocytopenia was reported.

This study will be the first prospective phase II trial to assess the pain efficacy and anti-tumor activity of Sn-117 m-DTPA in metastatic castration resistant prostate cancer with at least one clinically meaningful pain at baseline.

## Data Availability

Not applicable.

## Abbreviations

117 m-Sn-DTPA: Stannic(+ 4) -diethylenetriaminepentaacetic acid
AE/SEA: Adverse events/serious adverse events
ALP: Alkaline phosphatase
BPI-SF: Brief pain inventory-short form
CTCAE: Common Terminology Criteria for Adverse Events
CTEP: Cancer Therapy Evaluation Program
CTMS: Clinical Trials Monitoring Service
FDA: Food and Drug Administration
LHRH: Luteinizing hormone-releasing hormone
Mcrpc: Metastatic castration-resistant prostate cancer
ORR: Overall response rate
OS: Overall survival
PCWG3: Prostate Cancer Working Group 3
PFS: Progression-free survival
PRO-CTCAE: Patient Reported Outcomes for CTCAE
PSA: Prostate-specific antigen
SBRT: Stereotactic body radiation therapy
WHO: World Health Organization
